# Underestimated Effect Sizes in GWAS: Fundamental Limitations of Single SNP Analysis for Dichotomous Phenotypes

**DOI:** 10.1371/journal.pone.0027964

**Published:** 2011-11-28

**Authors:** Sven Stringer, Naomi R. Wray, René S. Kahn, Eske M. Derks

**Affiliations:** 1 Department of Psychiatry, Rudolf Magnus Institute of Neuroscience, University Medical Center Utrecht, Utrecht, The Netherlands; 2 Psychiatric Genetics Laboratory, Queensland Institute of Medical Research, Brisbane, Australia; 3 Queensland Brain Institute, University of Queensland, Brisbane, Australia; University of Bristol, United Kingdom

## Abstract

Complex diseases are often highly heritable. However, for many complex traits only a small proportion of the heritability can be explained by observed genetic variants in traditional genome-wide association (GWA) studies. Moreover, for some of those traits few significant SNPs have been identified. Single SNP association methods test for association at a single SNP, ignoring the effect of other SNPs. We show using a simple multi-locus odds model of complex disease that moderate to large effect sizes of causal variants may be estimated as relatively small effect sizes in single SNP association testing. This underestimation effect is most severe for diseases influenced by numerous risk variants. We relate the underestimation effect to the concept of non-collapsibility found in the statistics literature. As described, continuous phenotypes generated with linear genetic models are not affected by this underestimation effect. Since many GWA studies apply single SNP analysis to dichotomous phenotypes, previously reported results potentially underestimate true effect sizes, thereby impeding identification of true effect SNPs. Therefore, when a multi-locus model of disease risk is assumed, a multi SNP analysis may be more appropriate.

## Introduction

Since the first GWA study in 2005[1], hundreds of GWA studies have been published, reporting more than 2000 associations[2]. However, despite large heritability estimates, relatively few associations have been reported for most complex traits. Moreover, associations found in GWA studies often explain only a small proportion of the phenotypic variation[3]. For example, although 71 independent loci have been identified as being associated with Crohn's Disease, they still account for only 23% of the estimated heritability[4]. GWA studies of psychiatric diseases show an even less favorable picture. For instance, schizophrenia has an estimated heritability of 80%[5], [6], but observed genetic variants currently account for less than 1% of the variance[7].

One explanation of the missing heritability is that complex diseases are caused by a large number of causal variants with small effect sizes. Odds ratios (OR) reported in GWA studies are typically small (i.e., a median OR of 1.33[8]). The many associations that are tested require a very low significance threshold to prevent an inflated genome-wide type I error. This reduces the probability of identifying SNPs with small effect size, unless sample sizes are large enough to achieve sufficient power to identify such SNPs. Using large combined datasets within scientific consortia has significantly increased power in GWA studies. Despite this increase in power, still only a small number of associated variants have been identified[3]. A second explanation of the missing heritability is that risk SNPs are correlated with unobserved causal genetic variants, since they are unlikely to be causal themselves[9]. The lower the correlation between an observed risk SNP and the unobserved causal variant, the smaller the estimated effect size of the risk SNP, resulting in less explained variance and hence decreased power. This decrease in power is most dramatic for rare variants (i.e., SNPs with minor allele frequencies less than 5% or even 1%) and these variants are less likely to be tagged by the genotyped SNPs.

The present study addresses a fundamental limitation of traditional GWA analysis of dichotomous phenotypes which provides an additional explanation for the difficulty in identifying effect SNPs and the missing heritability. By definition complex diseases are caused by numerous risk variants. However, as single SNP analysis only considers a single SNP at a time, other SNPs associated with disease can be considered omitted covariates. Gail et al.[10] proved in the context of generalized linear models that omitting covariates can result in asymptotically underestimated effect sizes, even in the absence of confounders. Confounders are (possibly omitted) covariates that are associated with other covariates or variables of interest. Gail et al. showed that only the linear-link and log-link functions produce asymptotically unbiased effect sizes in generalized linear regression, although the log-link function can produce asymptotically biased intercepts[10]. In the context of logistic regression, this underestimation effect reduces the efficiency of effect size statistics[11]. Neuhauss and Jewell[12] provided formulas to assess this bias for several common link functions, including the logit and probit link functions, which are most suitable for analyzing dichotomous phenotypes. In linear regression omitting covariates has no effect on the estimated effect size[11].

The underestimation effect of non-linear link functions can be best understood in terms of the statistical concept of collapsibility. Simpson[13] wrote a seminal paper on the surprising non-equivalence of conditional and marginal odds ratios, which has later been referred to as Simpsons's paradox[14], [15]. Given three dichotomous variables X, Y, and Z, he showed that even if the odds ratios between X and Y conditional on the value of Z are equal 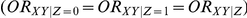, this does not imply that the marginal odds ratios equal the conditional odds ratio 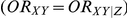. In other words, the odds ratio is a non-collapsible effect measure, as the marginal effect measure 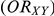 cannot generally be expressed as a weighted average of the conditional effect measures 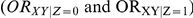. In the context of GWAS, Y is disease status, X is the genotype of an allele of interest, and Z is the number of risk variants in the genetic background. In this context Z is unlikely to be dichotomous. An effect size measure would be called collapsible if the marginal effect size of SNP X, averaged over all possible genetic backgrounds Z, can be expressed as a weighted average of all conditional effect sizes of SNP X (i.e., conditional on specific genetic background Z).[14], [15]

Two conditions have been identified that do result in collapsible odds ratios[16]. The first condition is that disease status Y and background Z are independent given SNP X. This implies that ignoring SNPs which have no effect on disease will not result in underestimation. The second condition is that SNP X and genetic background Z are independent given disease status Y. This situation cannot arise if we (safely) assume that SNPs or the causal variants with which they are in linkage disequilibrium cause disease status and not vice versa (see Hernán et al.[14] for a discussion on the importance of causal assumptions when dealing with Simpson's paradox). In other words, conditional and marginal odds ratios are only equivalent if the SNP of interest or the genetic background is not associated with disease status.

Despite the use of the word ‘bias’ by earlier authors[10]–[12], Greenland et al. [15] note that non-collapsibility is technically not a bias. It reflects the mathematical fact that for some effect measures marginal and conditional effect sizes are non-equivalent. When choosing a non-collapsible effect size measure, one merely needs to decide whether the marginal, the conditional effect size or both are of interest[14]. We believe that in GWA studies the odds ratio conditional on a fixed genetic background reflects the relative importance of a single SNP better than the marginal odds ratio. A single SNP analysis would estimate the marginal odds ratio, whereas a multi SNP analysis would estimate the odds ratio conditional on a fixed genetic background. Risk difference and risk ratio are examples of collapsible effect measures[15]. However, as traditional GWA analyses are often based on odds ratios, we will focus here on the logistic or odds disease model.

Complex diseases in GWA studies can be characterized by numerous risk SNPs with small effect sizes. Although the average effect size is expected to be small, the variance in the genetic background increases with the number of true risk SNPs. In the present simulation study we investigate the potential implications of non-collapsibility for traditional GWA studies. We first study the relation between the marginal and the conditional odds ratio under a naive disease model. The simplicity of the naive model facilitates the simulation and mathematical analysis of the underestimation effect. We report how disease characteristics (e.g., prevalence, number of risk SNPs, minor allele frequency, and effect sizes) influence the underestimation effect. We also show how this underestimation affects the estimated explained variance. Subsequently, we illustrate the underestimation effect under a more realistic genetic architecture. Finally, we discuss the implications of underestimating effect size and suggest potential solutions.

## Methods

**Figure 1:**
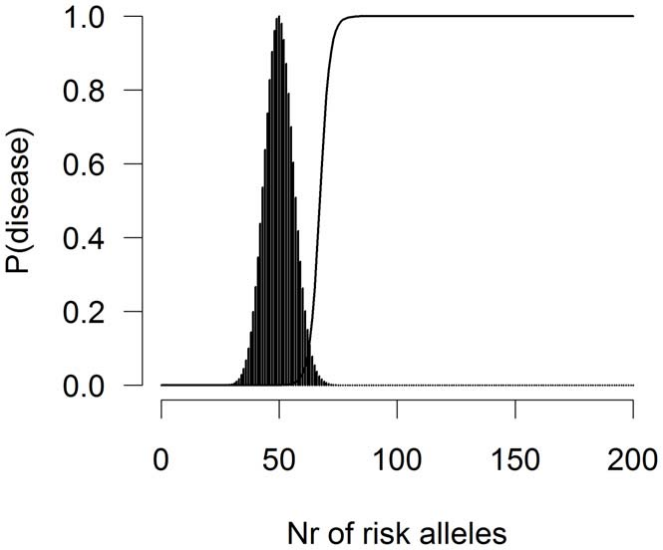
Disease model. Probability of disease as a function of the number of risk alleles (line) and the distribution of risk alleles in a large sample (n = 10,000) (histogram). Disease prevalence is 1%. The odds ratio of each risk SNP is 1.6 and the allele frequencies of risk alleles are 0.25. The maximum number of risk alleles is 200 (i.e., 100 SNPs). The (implicit) error variance of the odds model is 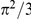.

Modeling a heritable disease requires a function relating genotype to disease risk. To simulate the implications of traditional GWA analysis using odds ratios, we constructed a disease generating model based on the odds model of disease risk. Before discussing this model in more detail, we illustrate the disease generating process of the odds model with an example. We assume that all risk alleles at different loci have equal frequency and equal effect size (these assumptions have been shown by others to have little impact on interpretation of results)[17]–[19]. For example, Figure 1 shows disease probability and the distribution of risk allele counts for a disease with a prevalence of 1%, assuming a total number of 200 effect alleles (i.e., 100 risk SNPs); the odds ratio of each risk allele is 1.6 and the risk allele frequencies are 0.25. Under this additive model on the log odds scale, people carry on average 50 risk alleles (binomial mean is 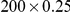) corresponding to a negligible disease risk. However, as the number of risk alleles exceeds a threshold, disease probability increases rapidly, demonstrating the highly non-linear relationship between genetic risk factors and disease risk. Those at highest risk of disease carry more risk alleles, 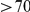 in this example, but each affected person could have a unique portfolio of risk alleles; the effect of a risk allele on disease depends on the genetic background (other risk alleles) carried by an individual. The (implicit) error variance in the odds model is 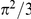, the variance of the standard logistic distribution.

As mentioned before, the marginal odds ratio produced by single SNP analysis is averaged over all possible genetic backgrounds. However, the odds ratios from the odds model, which we assume generates the disease, are conditional on a fixed background odds of disease (see section A3.2 in Appendix S1). We will therefore refer to the conditional odds ratio as the true odds ratio 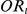 and to the marginal odds ratio as the (possibly under-)estimated odds ratio 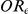. To relate 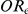 to the prespecified 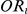, we performed the following four steps: (1) we specified a disease generating model based on disease characteristics including 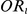, (2) we mathematically derived the genotype distribution of a single SNP of interest given disease status and disease characteristics, (3) we repeatedly simulated a case-control sample of the SNP of interest based on this genotype distribution and computed the corresponding SNP-based odds ratio 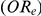, and (4) we reported the median of all estimated odds ratios 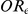, reflecting the asymptotic marginal odds ratio estimated by single SNP analysis. We now discuss the disease generating model.

We specified a disease generating model with four parameters: (1) disease prevalence 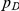, (2) true (allelic) odds ratio 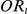, (3) minor allele frequency of risk alleles 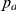, and (4) the total number of effect alleles 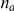. Risk alleles can be either minor alleles or major alleles. We only consider minor risk alleles, as the analysis is analogous for major risk alleles. Let 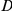 be disease status and 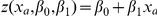 a linear function of the number of risk alleles 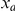 with effect size 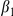 and intercept 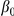. Then the probability of disease conditional on the number of risk alleles is defined as (see also Equation S3 in Appendix S1)
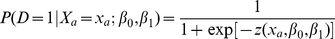
(1)


As effect size 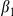 is defined on a log odds scale, 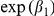 is the effect size on an odds scale. Therefore 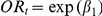 is the true odds ratio in the biological reality we aim to model.

So far we specified the probability of disease conditional on the number of risk alleles. To obtain a full probability model of disease, it is necessary to specify the distribution of risk alleles as well. Assuming Hardy-Weinberg equilibrium and linkage equilibrium for a total of 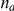 effect alleles (i.e., twice the number of risk SNPs) and risk allele frequency 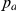, the number of risk alleles 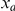 in the population can be modeled with a binomial distribution
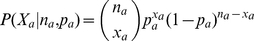
(2)


Combining distribution 1 and 2 results in a joint probability distribution of disease and number of risk alleles given four disease parameters: risk allele frequency 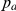, total number of effect alleles 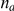, effect size 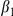 on a log odds scale, and intercept 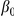.
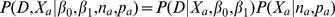
(3)


The probability of disease status 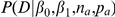 can be obtained by summing over all possible genetic liabilities 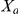.

Although 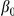 has an interpretation as the baseline (or background) log odds of disease, there is no strong prior information what this might be, as there is for the other three model parameters. However, as disease prevalence is an observed disease characteristic, it is possible to set 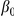 such that the disease probability of the model 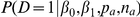 equals disease prevalence 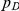. Although 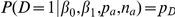 cannot be solved analytically for 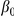, an error function, such as the sum squared error can be defined (Equation S1 in Appendix S1). This error function can be minimized to obtain a numerical approximation of 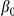 that satisfies the equality. Because 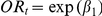 and number of risk SNPs 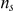 = 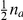, the result is a model of disease with the four parameters: disease prevalence 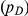, true allelic odds ratio 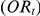, number of risk SNPs 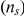, and risk allele frequency in risk SNPs 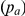. As a fifth parameter, error variance on the liability trait could be included to model the proportion of variance explained by all SNPs (heritability), but as this was not required for the derivations in this paper, we left the error variance implicit and constant (see section A3.4 in Appendix S1). From the four-parameter disease model we derived the genotype distribution of SNP 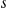 given disease status and model parameters 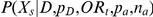 (see Equation S2 in Appendix S1). Based on this distribution we simulated 10,000 case-control samples and computed the median estimated SNP-based odds ratio 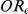. By relating the odds ratio 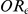 obtained when performing a single SNP analysis to the true odds ratio 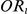, we could study the underestimation effect for different disease characteristics. Further details on simulation technicalities can be found in section A1 of Appendix S1.

Although the odds model is mathematically convenient, it assumes a constant effect size and minor allele frequency for all risk alleles. Therefore we performed a second simulation investigating the underestimation effect under a more realistic genetic architecture. In GWA studies absolute effect sizes on the log odds scale are roughly exponentially distributed[20], [21]. Consequently, effect sizes were drawn from an exponential distribution with rate parameter 5. This corresponds with an expected 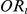 of 1.25, but acknowledges that true effect sizes are frequently small and rarely large. To avoid rare variants, allele frequencies were assumed to be uniformly distributed between 0.05 and 0.95. Effect sizes and allele frequencies were drawn once and fixed in the rest of the simulation replicates. The odds disease model from the first simulation is easily extended to accommodate different fixed effect sizes by defining 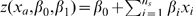 in Equation 1, where 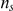 is the number of SNPs, 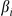 is the effect size of SNP 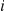 and 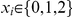 refers to the number of risk alleles at SNP 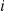. The intercept 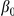
was chosen corresponding to a disease prevalence of 1%.

The asymptotic single SNP estimate was again assessed by generating 10,000 case-control samples and computing for each SNP the median odds ratio using a single SNP logistic regression. Case-control samples, 5000 subjects each, were generated by repeatedly drawing from the population distribution until 2500 cases and 2500 controls were sampled.

## Results

**Figure 2:**
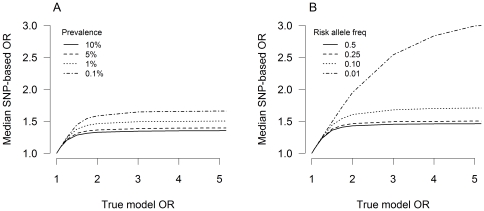
Numerous risk SNPs. Relationship between median estimated SNP-based odds ratio 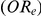 and true conditional model odds ratio 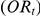 for a disease with 100 effect SNPs. (A) Different prevalences with risk allele frequency 0.25. (B) Different risk allele frequencies with prevalence 1%. Simulations are based on a case-control study of 3500 subjects and a 1∶1 case:control ratio. Medians are based on 10,000 case-control samples.

If a disease is caused by a single risk SNP, the odds ratio estimated by single SNP analysis (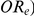 will, on average, reflect the true odds ratio (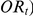 (section A2 in Appendix S1). However, if a disease is caused by numerous risk SNPs, the median 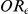 follows an asymptote. Figure 2 shows the relationship between median 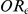 and 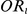 for diseases caused by 100 risk SNPs with different prevalences (A) and different minor allele frequencies of the risk SNPs (B). A wide range of prevalences and minor allele frequencies results in upper limits for the median SNP-based odds ratio. This asymptotic effect is more dramatic in diseases with higher prevalences and/or higher minor allele frequencies. Depending on the model parameters, the upper bound is reached with true model odds ratios as low as 1.5. In that case traditional association testing cannot differentiate, for example, between a true odds ratio of 1.5 and a true odds ratio of 3, as both will be estimated at 1.5, the maximum value that can be obtained. In other words, under this disease model large true effect sizes are not identified as such by single SNP association testing.

The asymptotic constraint on the estimated odds ratio is caused by two factors. First, single SNP odds ratios 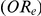 are estimated across an average over all possible background risks in cases and controls; this can be seen when computing the conditional probability of disease status given the genotype at a particular SNP (section A3.1 in Appendix S1). Only when the risk allele frequency 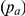 approaches zero, the background risk will approach zero, which is similar to a disease with a single risk SNP. This is why low risk allele frequencies (for example, *p_a_* = 0.01) result in a delayed asymptotic effect compared to high risk allele frequencies 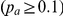 (Figure 2B). If the odds ratio for an allele could be estimated in a subsample of the population that all carried the same background risk, then the SNP-based odds ratio 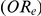 would (almost) equal the true odds ratio 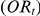 (see section A3.2 in Appendix S1).

Although weighted averaging is part of the explanation of the constrained odds ratios, it is not a sufficient explanation, because for continuous phenotypes the asymptotic effect does not occur when computing SNP-based effect sizes (section A3.3 in Appendix S1). It is due to the non-collapsibility of the odds ratio that averaging over background risks results in a discrepancy between the estimated marginal odds ratio 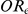 and the true conditional odds ratio 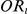.

**Figure 3:**
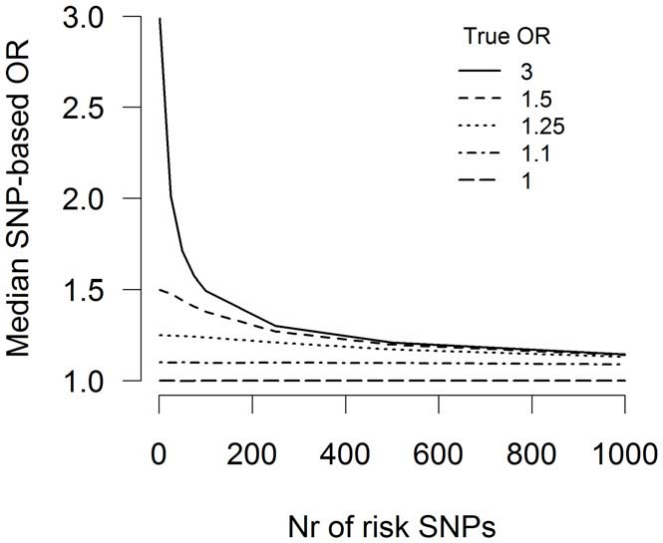
Number of risk SNPs. Effect of total number of risk SNPs on median SNP-based odds ratio 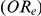 for different true odds ratios 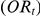. An allele frequency of 0.25 for risk alleles and a prevalence of 1% is assumed. Simulation is based on a sample of 3500 subjects and a 1∶1 case:control ratio. Median is based on 10,000 case-control samples.

A priori the total number of risk SNPs in a disease is unknown, but it is of course possible to simulate the results of traditional association testing for diseases with different numbers of risk SNPs. The asymptotic effect is stronger for diseases which are influenced by a large number of risk SNPs (Figure 3). In other words, an increase in the number of SNPs associated with disease results in increased underestimation. As complex diseases are assumed to be influenced by many risk SNPs, analyzing numerous large-effect SNPs with traditional association testing would result in considerable underestimation. This type of underestimation is not due to a lack of power as increasing sample size will decrease the variance of effect sizes obtained, but will not reduce underestimation due to the non-collapsibility of the odds ratio.

**Figure 4:**
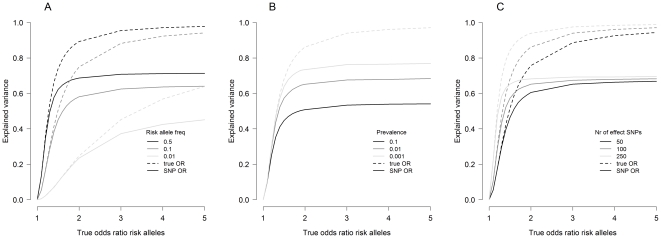
Explained Variance. McKelvey-Zavoina's 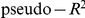 (on log odds scale) as a function of true effect size for an odds model with true odds ratio (dashed line) and an odds model with median odds ratio obtained by single SNP analyses (solid line) for (A) different risk allele frequencies, (B) different prevalences, and (C) different number of effect SNPs. Unless stated otherwise models are based on a disease prevalence of 1%, 100 effect SNPs with risk allele frequencies of 0.25, a case-control sample of 3500 subjects and a 1∶1 case:control ratio.

We will now show that underestimation of effect sizes can result in additional missing heritability. Narrow-sense heritability is the percentage of total phenotypic variance that is explained by additive genetic variance. Figure 4 compares the explained variance (on the log odds scale) of true odds models with the explained variance based on effect sizes obtained from single SNP association tests. Although many measures of explained variance exist for logistic regression, we adopted McKelvey-Zavoina's 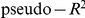 [22], as it is defined on the log odds scale and closely mirrors the explained variance of continuous traits [23](see section A3.4 in Appendix S1 for more details on McKelvey-Zavoina's 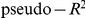).

McKelvey-Zavoina's 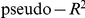 strongly depends on the effect size of risk alleles and the genetic variance in risk SNPs. Therefore even true odds models show little explained variance in case of small effect sizes or low minor allele frequencies 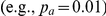. Except for diseases with rare causal variants, true models with moderate to large effect sizes explain more than 80% of total variance, approaching 100% for very large effect sizes, indicating large heritability. However, odds models based on underestimated SNP-based odds ratios 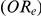 show a loss in explained variance compared to odds models based on true effect sizes 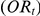. In the unrealistic case of 100% heritability the typical loss of explained variance is around 20%. A more realistic disease with a heritability of 80%, prevalence of 1% and a minor allele frequency of 50%, still results in an expected loss of more than 10% in explained variance (see Figure 4A). Although prevalence does not affect the true heritability (dotted line), it does affect the heritability based on 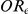
(solid line) (Figure 4B).

Truly associated SNPs are unknown a priori and effect sizes will be estimated with error. Nonetheless, this analysis shows that even if truly associated SNPs are known and effect sizes are estimated without error, traditional association testing on dichotomous phenotypes can result in a significant loss of explained variance.

**Figure 5:**
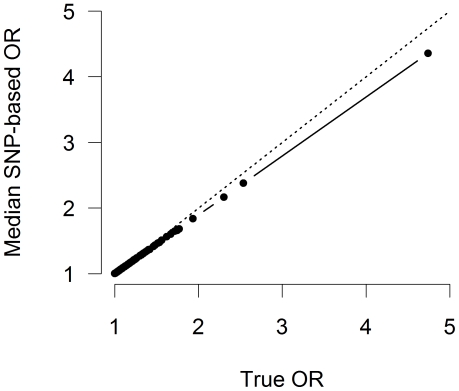
Varying effect sizes. Relationship between median estimated SNP-based odds ratio 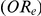 and true conditional model odds ratio 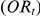 for a disease with 100 effect SNPs and a disease prevalence of 1%. Effect sizes on log odds scale were drawn once for each SNP from an exponential distribution with rate parameter 5 and fixed for all 10,000 case-control simulations. Similarly, allele frequencies were drawn once for each SNP from a uniform distribution between 0.05 and 0.95 and fixed for all case-control simulations. Case-control simulation was based on a sample of 5000 subjects and a 1∶1 case:control ratio.

The previous results were all based on the assumption of fixed effect size and allele frequency. Figure 5 shows odds ratios estimated with single SNP analysis, using a more realistically simulated data set in which absolute effect sizes are exponentially distributed and minor allele frequencies are uniformly distributed. Moderate and large odds ratios are underestimated and the underestimation effect increases with effect size. For example the highest risk SNP with a true (conditional) odds ratio of 4.74 has a marginal odds ratio of 4.36, resulting in underestimation of 9% on the odds scale. As expected, odds ratios close to one do not show underestimation. Similar to the naive disease model results, increasing the average true odds ratio, the number of effect SNPs, or the prevalence further increases the underestimation effect (data not shown).

## Discussion

Summarizing, our analysis shows a fundamental limitation of applying single SNP association tests to dichotomous phenotypes. Single SNP tests can severely underestimate moderate and large effect sizes for diseases with numerous risk SNPs due to non-collapsibility of the odds ratio. Therefore the marginal odds ratios obtained by single SNP tests can be smaller than the true conditional odds ratios. This underestimation reduces the explained variance and hence contributes to the missing heritability. Underestimation is most pronounced in diseases with high-risk SNPs (i.e., mean 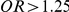), common affect SNPs (i.e., 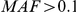), a large number of risk SNPs (i.e., 100 or more) and high prevalence 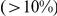.

Our results are consistent with empirical findings in the GWAS literature. Odds ratios reported in GWA studies are generally small[8]. For example, a recent GWA study reported 57 regions outside the major histocompatibility complex associated with multiple sclerosis, none of which had an odds ratio much higher than 1.5 (see Figure 2 in[24]). Although occasionally large effect sizes have been reported, numerous common high-risk SNPs have not been identified for a single dichotomous trait. Searching the GWAS catalogue (http://www.genome.gov/gwastudies; accessed August 24, 2011) for SNPs with 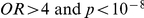, shows that no single study reports a disease that is influenced by two or more common SNPs with 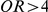. Diseases for which high odds ratios are reported for common SNPs (with minor allele frequency in controls 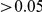) include auto-immune diseases such as type I diabetes 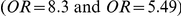 [25], [26] and ciliac disease 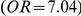 [27]. These high-risk SNPs are part of the major histocompatibility complex.

Single SNP analysis cannot identify large effect sizes of numerous risk SNPs, even if many high risk SNPs would exist. This scenario is mostly of theoretical interest though, as research on quantitative traits, which are not affected by non-collapsibility, suggests that numerous high risk SNPs are not likely in practice. However, conditional odds ratios are likely to be larger than the marginal odds ratios commonly reported. The significance thresholds for marginal and conditional odds ratio are equal as both odds ratios are equivalent in case of no effect[15]. That is, under the null distribution underestimation is not an issue. Therefore, underestimation impedes the identification of SNPs above the significance threshold with underestimated values below the significance threshold.

GWA studies of diseases with high prevalence have reported less significantly associated genetic variants than similar studies of diseases with low prevalence. For example, GWA studies of major depression disorder, which has a life time prevalence of 15%, have reported no associations that reached genome-wide significance or have been solidly replicated[7], [28]. On the other hand, studies of schizophrenia and bipolar disorder, which have life time prevalences of 1% or less, have reported several SNPs that did reach genome-wide significance and/or were replicated[7]. There are likely to be many factors contributing to the differential success of GWAS for psychiatric disorders. For example, a lower heritability for depression compared to schizophrenia could imply smaller effect sizes under an architecture of the same number of causal variants, hence requiring larger sample sizes to achieve the necessary power to detect variants that explain the same proportion of variance. Nonetheless, the empirical data are consistent with our result that the underestimation of effect size is larger and the explained variance in liability is lower for complex diseases with high prevalence compared to diseases with low prevalence.

The underestimation effect due to non-collapsibility has important implications for GWA studies of complex diseases. An important aim of GWA analyses is to select truly associated SNPs for use in subsequent analyses and to identify causal variants[19], [29]. For selection purposes moderate underestimation of effect sizes need not be a problem, if sample sizes are large enough. However, underestimation of effect size requires larger sample sizes to identify both truly associated SNPs and causal variants. One solution to avoid underestimation of true effect sizes is to analyze continuous instead of dichotomous phenotypes, if available. Continuous phenotypes can usually be modeled with linear regression and under an additive genetic model SNPs are independent and single SNP association tests will not result in underestimation. The use of continuous phenotypes is consistent with the quest for endophenotypes for complex (psychiatric) diseases[30]. Another solution is to estimate effect sizes of all SNPs simultaneously rather than individually. It is for example feasible to estimate the effect sizes of more than 100,000 SNPs in a single analysis[31]. Based on the results of Robinson et al.[11], we expect that a multi SNP analysis is more powerful than a single SNP analysis in the context of a complex disease. Methods for estimating aggregate statistics such as explained variance, total number of risk SNPs, and average effect size of risk SNPs, which analyze all SNPs simultaneously, also exist[32]–[34]. Even in the context of continuous traits it might be beneficial to opt for multi SNP analysis, as adding covariates can reduce the standard error of the estimates, requiring a smaller sample size to achieve significance.

There are some limitations to our analysis. First of all, our conclusions are conditional on simple model assumptions. However, simpe assumptions do underscore the fundamental nature of the underestimation effect. A second limitation is that we have not proved that effect sizes reported in traditional GWA studies are indeed underestimated. Biases such as the winner's curse could also result in overestimation[35], [36]. The winner's curse refers to the fact that due to stringent multiple testing correction it is likely that the first significant finding of a SNP will have a larger effect size than subsequent independent replications. It is therefore unclear whether in practice reported odds ratios are overestimated or underestimated. The major difference between the underestimation effect we discuss and the winner's curse bias, is that the latter will decrease as the sample size increases, whereas non-collapsibility results in a fundamental underestimation that is not affected by sample size. Finally, although we show that underestimation can partly explain missing heritability, this effect could be modest. Continuous traits such as human height are not affected by the underestimation effect, but also show missing heritability[37].

In conclusion, single SNP association testing on dichotomous phenotypes can be problematic. Our analysis implies that odds ratios typically reported in GWA studies [8] could be underestimates of the true conditional odds ratios. We argue that asymptotic underestimation is a serious draw-back, as it cannot be remedied by increasing sample size. We therefore recommend analyzing all SNPs simultaneously. As a variety of multi SNP methods have been proposed in the literature, we are currently comparing the performance of several of those on real GWAS data.

## Supporting Information

Appendix S1 **Supplemental Appendix.**
(PDF)
