# Supplementary material for: Underestimated Effect Sizes in GWAS: Fundamental Limitations of Single SNP Analysis for Dichotomous Phenotypes
Source: PLoS One. 2011 Nov 28;6(11):e27964. doi: 10.1371/journal.pone.0027964 (PMC3225388; doi:10.1371/journal.pone.0027964)
Supplement: Appendix S1 — Supplemental Appendix. (PDF) [file pone.0027964.s001.pdf]

S1 Supplemental Appendix

A1 Simulation details

To simulate a distribution of estimated SNP-based allelic odds ratio OR_e, we computed the odds ratio from 10,000 simulated 2x2 allelic cross tabulations at one risk SNP (risk/protective allele versus case/control). As effect sizes and risk allele frequencies were assumed to be equal for all risk SNPs, risk SNPs were interchangeable and hence shared the same distribution of OR_e. The allelic cross tabulations were simulated by drawing from a categorical distribution with the average relative cell frequencies of the cross tabulations as parameters. Average relative frequencies were derived analytically from the model parameters in the disease model. As each SNP consists of two alleles, the sample size in each simulated cross tabulation was twice the number of subjects in the case-control study. All simulations were based on a study sample of 3500 subjects with a 1:1 case:control ratio.

The average relative cell frequencies in a 2x2 allelic cross tabulation at a particular SNP in a case-control study can be expressed as a sample dependent joint probability $P_{sample}(X_a, D)$ of allele X_a and disease status D (risk allele a : $X_a = 1$, protective allele A : $X_a = 0$; case: $D = 1$, control: $D = 0$). As only genotypes are observed $P_{sample}(X_a, D)$ needs to be derived from a similar distribution $P_{sample}(X_s, D)$ based on a 3x2 genotype cross tabulation ($X_s \in \{0 = AA, 1 = Aa, 2 = aa\}$ is the number of risk alleles in that particular SNP). Table S1 shows the allelic joint probability as a function of genotype-based joint probabilities in a case-control sample. Note that heterozygosity contributes half to the a row and half to the A row in the allelic cross tabulation. We then derived $P_{sample}(X_s, D)$. Because disease status D is known for all subjects in a case-control study, the joint distribution of the genotype cross table can be written as $P_{sample}(X_s, D) = P(X_s|D)P_{sample}(D)$. $P(X_s|D)$ is the probability of genotype X_s given disease status D. As $P_{sample}(D)$ is the percentage of cases in the case-control sample, the joint distribution is sample dependent. For simplicity, we have assumed case-control samples with a 1:1 case:control ratio. Note that the sample joint distribution $[P_{sample}(X_s, D)]$ does not equal the joint distribution in the population $[P(X_s, D)]$, as the percentage of cases in a case-control study $[P_{sample}(D = 1)]$ typically does not equal the prevalence in the population $[P(D = 1)]$.

Allelic cross tabulation

	D	$\overline{D}$
a	$P_{sample}(aa, D) + \frac{1}{2}P_{sample}(Aa, D)$	$P_{sample}(aa, \overline{D}) + \frac{1}{2}P_{sample}(Aa, \overline{D})$
A	$P_{sample}(AA, D) + \frac{1}{2}P_{sample}(Aa, D)$	$P_{sample}(AA, \overline{D}) + \frac{1}{2}P_{sample}(Aa, \overline{D})$

Table S1. Allelic joint probabilities as a function of genotype-based joint probabilities in case-control sample.

Finally, we analytically derived the conditional probability of a genotype at a particular SNP given disease status as a function of the four disease model parameters: $P(X_s|D, \beta_0, \beta_1, p_a, n_a)$ (for more details on the mathematical analysis we refer to section A3 below).

The simulation procedure described above allowed efficient simulation of SNP-based odds ratio OR_e values for risk SNPs based on disease prevalence, true effect size, risk allele frequency,

and total number of risk SNPs. By simulating distributions of OR_e for a plausible range of model parameter values we were able to study the relationship between median OR_e and true model odds ration OR_t.

For a given disease prevalence, we set the intercept β_0 such that the probability of disease in the population matches the predefined disease prevalence. Let D be disease status, X_a the number of risk alleles an individual caries, $\beta_1 = \log(OR_t)$ the effect size on log odds scale, p_a the risk allele frequency, and n_a the total number of effect alleles, i.e. twice the number of risk SNPs, and β_0 the intercept such that $P(D = 1|\beta_0, \beta_1, p_a, n_a) = p_D$. Given disease prevalence p_D and assuming Hardy-Weinberg equilibrium, the intercept was computed by numerically approximating the minimum of the following squared error function.

$$
\begin{aligned}
f(\beta_0) &= (P(D = 1|\beta_0, \beta_1, p_a, n_a) - p_D)^2 \\
&= (\sum_{x_a=0}^{n_a} P(D = 1|X_a = x_a, \beta_0, \beta_1)P(X_a = x_a|p_a, n_a) - p_D)^2 \\
&= (\sum_{x_a=0}^{n_a} \frac{\binom{n_a}{x_a} p_a^{x_a}(1-p_a)^{n_a-x_a}}{1+\exp(-\beta_0 - \beta_1 x_a)} - p_D)^2
\end{aligned} \quad \text{(S1)}
$$

A2 Relationship between marginal odds ratio OR_e and conditional OR_t for diseases with single effect SNP

Figure S1 shows the relationship between median estimated odds ratio OR_e and true conditional odds ratio OR_t for diseases with a single risk SNP, different prevalences (A), and different minor allele frequencies (B). Ideally, the median estimated effect size of SNPs obtained from single SNP association testing should reflect the true effect size OR_t of the disease generating model. As Figure S1 shows, for $OR_t < 3$ the median OR_e reflects OR_t fairly well. For larger true odds ratios we found a non-linear relationship between OR_e and OR_t, depending on prevalence and risk allele frequency. This is due to the fact that the true conditional odds ratio OR_t is based on a single allelic position, whereas the estimated SNP-based odds ratio OR_e is based on two allelic positions. We refer to section A3.2 below for mathematical details.

A3 Mathematical Analysis

A3.1 Deriving probability of SNP genotype given disease status

The joint distribution genotype-based cross tables per SNP used in the simulation ignore background risk, as they only involve a single risk SNP. However, the odds model is specified conditional on the total number of minor risk alleles a subject caries. It is therefore necessary to average over all possible genetic backgrounds to compute the probability of a genotype at SNP s given disease status. Combining this with Bayes rule we obtain the following analytical derivation.

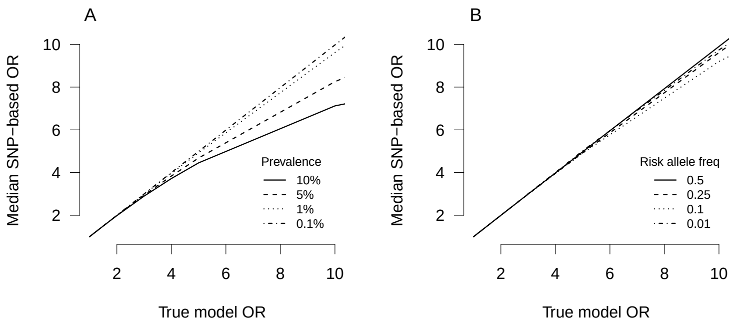

Figure S1. Single risk SNP. Relationship between median SNP-based odds ratio (OR_e) and true model odds ratio (OR_t) for disease with single risk SNP. (A) Different prevalences with minor allele frequency 0.25. (B) Different risk allele frequencies with prevalence 1%. Simulations are based on case-control study of 3500 subjects and 1:1 case:control ratio. Medians based on 10,000 case-control samples.

$$
\begin{aligned}
P(X_s|D,\beta_0,\beta_1,p_a,n_a) &= \frac{P(D|X_s,\beta_0,\beta_1,p_a,n_a)P(X_s|p_a)}{P(D|\beta_0,\beta_1,p_a,n_a)} \\
&= \frac{\left[\sum_{x_{bg}=0}^{n_a-2} P(D|X_s,x_{bg},\beta_0,\beta_1)P(x_{bg}|p_a,n_a)\right] P(X_s|p_a)}{P(D|\beta_0,\beta_1,p_a,n_a)} \\
&= \frac{\left[\sum_{x_{bg}=0}^{n_a-2} \frac{\binom{n_a-2}{x_{bg}} p_a^{x_{bg}}(1-p_a)^{n_a-x_{bg}}}{1+\exp(-\beta_0-\beta_1(X_s+x_{bg}))}\right] \left[\binom{2}{x_s} p_a^{X_s}(1-p_a)^{2-X_s}\right]}{\left[\sum_{x_a=0}^{n_a} \frac{\binom{n_a}{x_a} p_a^{x_a}(1-p_a)^{n_a-x_a}}{1+\exp(-\beta_0-\beta_1 x_a)}\right]^D \left[1-\sum_{x_a=0}^{n_a} \frac{\binom{n_a}{x_a} p_a^{x_a}(1-p_a)^{n_a-x_a}}{1+\exp(-\beta_0-\beta_1 x_a)}\right]^{1-D}}
\end{aligned}
\qquad \text{(S2)}
$$

The final expression comprises a quotient of four terms (defined by square brackets). The first term in the numerator is the probability of disease given the genotype at a particular SNP. The second term in the numerator is the probability of the genotype at that particular SNP given the risk allele frequency. The two terms in the denominator are the probability of disease and non-disease in the population.

A3.2 Approximate equivalence of conditional allelic odds ratio and marginal SNP-wise odds ratios under a single SNP model

To clarify the relationship between the estimated SNP-based odds ratio OR_e and the true odds ratio OR_t, we introduce the notion of allelic odds ratio. An allelic odds ratio OR_a is the odds ratio of an extra risk allele versus no extra risk allele given a fixed background odds of disease.

For a (sub)population with no variation in background odds of disease, it is possible to show that the allelic odds ratio OR_a at a particular allele equals the true odds ratio OR_t.

Let $X_s \in \{0, 1, 2\}$ be the number of risk alleles at a particular SNP, and X_{bg} the number of risk alleles in the remaining background SNPs. Then $X_a = X_s + X_{bg}$ is the total number of risk alleles. Note that

$$\begin{aligned} P(D = 0|X_a = x_a, \beta_0, \beta_1) &= 1 - P(D = 1|X_a = x_a, \beta_0, \beta_1) \\ &= \frac{\exp[-(\beta_0 + \beta_1 x_a)]}{1 + \exp[-(\beta_0 + \beta_1 x_a)]} \\ &= \frac{1}{\exp(\beta_0 + \beta_1 x_a) + 1} \end{aligned} \tag{S3}$$

We use this equality to demonstrate that the allelic odds ratio OR_a for people with the same genetic background x_{bg} equals the true odds ratio $OR_t = \exp(\beta_1)$. The fifth equality below is obtained by multiplying both the numerator and the denominator by $\exp[\beta_0 + \beta_1(1 + x_{bg})]$.

$$\begin{aligned} OR_a &= \frac{P(D = 1, X_s = 1|X_{bg} = x_{bg})P(D = 0, X_s = 0|X_{bg} = x_{bg})}{P(D = 0, X_s = 1|X_{bg} = x_{bg})P(D = 1, X_s = 0|X_{bg} = x_{bg})} \\ &= \frac{P(D = 1|X_s = 1, X_{bg} = x_{bg})P(X_s = 1)P(D = 0|X_s = 0, X_{bg} = x_{bg})P(X_s = 0)}{P(D = 0|X_s = 1, X_{bg} = x_{bg})P(X_s = 1)P(D = 1|X_s = 0, X_{bg} = x_{bg})P(X_s = 0)} \\ &= \frac{P(D = 1|X_a = 1 + x_{bg})P(D = 0|X_a = x_{bg})}{P(D = 0|X_a = 1 + x_{bg})P(D = 1|X_a = x_{bg})} \\ &= \frac{(1 + \exp[\beta_0 + \beta_1(1 + x_{bg})])(1 + \exp[-(\beta_0 + \beta_1 x_{bg})])}{(1 + \exp[-(\beta_0 + \beta_1(1 + x_{bg}))])(1 + \exp[\beta_0 + \beta_1 x_{bg}])} \\ &= \frac{(1 + \exp[\beta_0 + \beta_1(1 + x_{bg})])(\exp[(\beta_0 + \beta_1(1 + x_{bg}))] + \exp(\beta_1))}{(1 + \exp[\beta_0 + \beta_1 x_{bg}])(\exp[(\beta_0 + \beta_1(1 + x_{bg}))] + 1)} \\ &= \exp(\beta_1) \\ &= OR_t \end{aligned} \tag{S4}$$

This equality demonstrates theoretically that we could estimate OR_t by computing OR_a at a particular SNP (i.e., aA versus AA or aa versus Aa) in a subset of the population carrying the same genetic background risk x_{bg} for any x_{bg}. Due to variation in background risk within a population equality $OR_t = OR_a$ will generally not hold in a study sample. Only for diseases with a single effect SNP the background risk is equal for all subjects (i.e., no background risk) and hence the equality will hold.

Note that the definitions of OR_a and OR_t are based on a single allelic position, whereas GWA studies often report SNP-based allelic odds ratios (OR_e), based on two allelic positions. As $OR_a = OR_t$ for diseases with a single risk SNP, Figure S1 shows the discrepancy between SNP-based estimate OR_e and allele-based estimate OR_a for different model parameters. Although OR_e and OR_a are not equal, their difference is small compared to the underestimation of effect size in complex diseases.

A3.3 Equivalence of marginal and conditional effect size under a linear regression model

Let $y = \beta_0 + \beta_1 x_a + \epsilon$ be the value of continuous phenotype Y as a function of the number of risk alleles x_a. The expected value of phenotype Y given genotype X_s and background X_{bg} is given by the linear model. Because the background is unknown when analyzing a particular risk SNP, it is necessary to average over all possible backgrounds by computing the expected value once again. Hence the expected value of phenotype Y given genotype x_s is

$$
\begin{aligned}
E(Y|x_s) &= E[E(Y|x_s, X_{bg})] \\
&= E[\beta_0 + \beta_1(x_s + X_{bg})] \\
&= \beta_0 + \beta_1(x_s + E[X_{bg}]) \\
&= \beta_0 + \beta_1(x_s + (n_a - 2)p_a)
\end{aligned}
\tag{S5}
$$

As before, n_a is the total number of effect alleles and p_a the risk allele frequency. The average SNP-based effect size B_s is the difference in average phenotype of Aa genotypes (Y_{Aa}) and AA genotypes (Y_{AA}) at risk SNP s. Therefore the expected effect size obtained at SNP s is

$$
\begin{aligned}
E(B_s|X_s) &= E(Y_{Aa} - Y_{AA}) \\
&= E(Y_{Aa}) - E(Y_{AA}) \\
&= E(Y|X_s = 1) - E(Y|X_s = 0) \\
&= (\beta_0 + \beta_1(1 + (n_a - 2)p_a)) - (\beta_0 + \beta_1(0 + (n_a - 2)p_a)) \\
&= \beta_1
\end{aligned}
\tag{S6}
$$

Hence single SNP association testing on continuous disease traits under a linear model can, on average, identify the true conditional effect sizes.

A3.4 Effect of underestimation on explained variance

In linear regression R^2 can be computed to assess the explained variance of a genetic model. For logistic regression, many pseudo$-R^2$ measures exist without agreement on the measure that should be applied. We adopted McKelvey-Zavoina's pseudo $- \mathrm{R}^2$. [1] Simulation studies have shown that, compared to other pseudo $- \mathrm{R}^2$ statistics, it produces results that are most similar to the R^2 obtained when applying linear regression to an observed liability trait. [2]

McKelvey-Zavoina's pseudo $- \mathrm{R}^2$ mirrors the explained variance in linear regression by assuming a normally distributed latent liability trait $Y^* = \beta_0 + \beta_1 X_a + \epsilon$ with $\epsilon \sim \mathcal{N}(0, \sigma_\epsilon)$. McKelvey-Zavoina's pseudo $- \mathrm{R}^2$ is the percentage of variance in latent liability trait explained by the model. The total disease trait variance Y^* consists of variance explained by the model

and the error variance of the (approximated) probit model. Let $\overline{Y}^* = \beta_0 + \beta_1 X_a$ be the latent trait as predicted by the model. Then the explained variance is

$$\begin{aligned} R^2_{\texttt{M-Z}} &= \frac{\mathrm{Var}(\overline{Y}^*)}{\mathrm{Var}(\overline{Y}^*) + \mathrm{Var}(\epsilon)} \\ &= \frac{\mathrm{Var}(\overline{Y}^*)}{\mathrm{Var}(\overline{Y}^*) + \sigma_\epsilon^2} \end{aligned} \tag{S7}$$

The variance of $\overline{Y}^*$ can be written as

$$\begin{aligned} \mathrm{Var}(\overline{Y}^*) &= \mathrm{Var}(\beta_0 + \beta_1 X_a) \\ &= \beta_1^2 \mathrm{Var}(X_a) \\ &= \beta_1^2 n p_a (1 - p_a) \end{aligned} \tag{S8}$$

The estimated explained variance is a function of coefficient β_1, number of effect alleles n_a, allele frequency p_a, and standard error σ_ϵ. The estimated effect size β_1 influences the variance of $\overline{Y}^*$ and hence the estimated explained variance. In other words, underestimated effect sizes result in an underestimated explained variance.

Although the choice of σ_ϵ does not affect the estimation of the logistic coefficients, it does affect the estimated explained variance. In the probit model $P(D = 1|X_a = x_a) = f_{\mathrm{probit}}(y* = \beta_0 + \beta_1 x_a)$, which implies that a $y* > 0$ will result in disease. To ensure that the probit model is identifiable, it is customary to set the standard error σ_ϵ to 1. The difference between the odds model and the probit model is the link function. That is, in the odds model $P(D = 1|X_a = x_a) = f_{logit}(\beta_0 + \beta_1 x_a)$. This link implies a logistic error term instead of a normal error term. Because McKelvey-Zavoina's pseudo $-$ R^2 is based on a normally distributed latent trait, $(15/16)(\pi/\sqrt{3}) f_{\mathrm{logit}}(y*) \approx f_{\mathrm{probit}}(y*)$ is a good approximation of the probit model. It is conventional to use the standard logistic distribution for the error term, which has a standard error of $\pi/\sqrt{3}$. However, a multiplication factor of $(15/16)(\pi/\sqrt{3})$ provides the best approximation to the probit model on which McKelvey-Zavoina's pseudo $-$ R^2 is based. [3]

References

1. McKelvey RD, Zavoina W (1975) A statistical model for the analysis of ordinal level dependent variables. J Math Sociol 4: 103–120.

2. DeMaris A (2002) Explained variance in logistic regression: A Monte Carlo study of proposed measures. Sociol Methods Res 31: 27–74.

3. Camilli G (1994) Origin of the scaling constant d = 1.7 in item response theory. J Educ Behav Stat 19: 293–295.
